# Diet Quality, Nutritional Adequacy and Anthropometric Status among Indigenous Women of Reproductive Age Group (15–49 Years) in India: A Narrative Review

**DOI:** 10.3390/dietetics2010001

**Published:** 2022-12-22

**Authors:** Ridhima Kapoor, Manisha Sabharwal, Suparna Ghosh-Jerath

**Affiliations:** 1 Lady Irwin College, University of Delhi, Sikandra Road, New Delhi 110001, India; 2 The George Institute for Global Health, New Delhi 110025, India

**Keywords:** dietary intake, food consumption, nutrient intake, nutritional status, diet quality, indigenous women, Indian tribal women

## Abstract

In India, indigenous communities are nutritionally vulnerable, with indigenous women suffering the greater burden. Studies and surveys have reported poor nutritional outcomes among indigenous women in India, yet systematic documentation of community-specific nutrition data is lacking. We conducted a narrative review of 42 studies to summarise the nutritional profile of indigenous women of India, with details on their food and nutrient intakes, dietary diversity, traditional food consumption and anthropometric status. Percentage deficits were observed in intake of pulses, green leafy vegetables, fruits, vegetables, flesh foods and dairy products when compared with recommended dietary intakes for moderately active Indian women. Indices of diet quality in indigenous women were documented in limited studies, which revealed poor dietary diversity as well as low consumption of diverse traditional foods. A high risk of nutritional inadequacy was reported in all communities, especially for iron, calcium, and vitamin A. Prevalence of chronic energy deficiency was high in most communities, with dual burden of malnutrition in indigenous women of north-eastern region. Findings from this review can thus help guide future research and provide valuable insights for policymakers and program implementers on potential interventions for addressing specific nutritional issues among indigenous women of India.

## 1 Introduction

Indigenous Peoples, who represent five percent of the global population, are amongst the most vulnerable and marginalized human populations in the world. They face extreme poverty in all spheres and dimensions, suffer from poor health and nutrition outcomes, and have a compromised quality of life than their non-indigenous counterparts [1]. India, a land of numerous cultures and people, is home to 104 million Indigenous Peoples, who constitute 8.6% of the total national population [2]. These Indigenous Peoples, identified as ‘Scheduled Tribes (STs)’ in India, constitute the second largest indigenous population in the world [3]. The Government of India identifies these STs or indigenous communities based on their traditional traits, distinctive culture, geographical isolation, and lack of progress on the social and economic front [4].

There are around 705 different Indian indigenous communities living in 15% of the country’s area, predominantly in forests, hills and inaccessible regions (including deserts and plain areas) [5]. Among these indigenous communities, 75 communities are identified as particularly vulnerable tribal groups (PVTGs) due to their pre-agricultural technology, declining or stagnant population, low literacy and dependence on subsistence economy [6]. Although, indigenous communities in India differ from one another in terms of their racial traits, dialects, socio-cultural customs, and patterns, they face common day-to-day challenges due to their location in geographically isolated areas and their dependence on natural ecosystems for basic livelihood [6]. They face discrimination than other social groups, in terms of economic development, social status, income distribution and access to basic health facilities [6,7]. Further, indigenous communities have access to diverse natural food sources that provide wild traditional foods that are native to a particular region and are rooted in social and cultural identities of indigenous populations [8–10]. However, despite possessing traditional ecological knowledge (TEK) about their unique local food systems, indigenous communities in India are nutritionally vulnerable [6]. Surveys conducted by National Nutrition Monitoring Bureau (NNMB) in nine states, have reported poor nutrient intakes among the indigenous population, particularly for essential micronutrients such as vitamin A, iron, and riboflavin [11]. Consumption of most food groups among these communities was found to be below the recommended dietary intake of Indians [12] and has reduced over the years, indicating the risk of rising food and nutrition insecurity among the indigenous population [6,11].

Although malnutrition is prevalent across all segments of the indigenous population in India, the group that gets most affected is indigenous women and children [7,13,14]. Indigenous women in India participate in all types of activities, including household work, agricultural work and child rearing, and contribute to the local economy by participating with men in subsistence activities [14–16]. According to the literature, indigenous women are considered an economic asset in the society, yet face greater disadvantage than their male counterparts in terms of food consumption and access [14,15]. A case study in a tribal district of Jharkhand revealed that female headed households are more vulnerable to nutritional insecurity, as they often face problems related to food starvation and food procurement [16], while two review studies have reported poor calorie intake among indigenous women of central-eastern India which falls short to compensate for their heavy physical workload [17,18]. A study by UNICEF found that indigenous women make up a disproportionately large number of maternal deaths in some states, which has been attributed to the high incidence of chronic energy deficiency (CED) and anemia among these women [19]. According to recent National Family Health Survey (NFHS)-5, 2019-21, about 25% of the indigenous women are chronically energy deficient (Body Mass Index (BMI) < 18.5 kg/m^2^) among which 15% are mildly thin and 10.6% are moderately/severely thin [20]. This is slightly higher than the national prevalence reported for CED in Indian women (18.7%), with 11% as mildly thin and 7% as moderately/severely thin [20]. The recent NFHS-5 data also suggests a wide gap in the prevalence of CED and anemia between indigenous men and women. All these factors might further contribute to overall poor nutritional status among indigenous women, who in the future, maybe likely to give birth to low-weight infants, thus leading to a vicious cycle of malnutrition.

Although studies and surveys have been carried out on dietary intake and nutritional status of indigenous women in India, there exists a near absence of community-specific nutrition data for indigenous women of reproductive age group. This is a crucial information gap as baseline nutritional data on non-pregnant indigenous women may aid in the development of future strategies to improve maternal and child health outcomes among indigenous communities of India. Further, there is lack of data on consumption of local traditional foods among women belonging to indigenous communities of India. The impact of seasonality on dietary intake of indigenous women have also not been explored to a desired extent. This could be a critical opportunity to research as changes in temperature, wind and rainfall patterns influence the crop production and wild food availability. This may have an impact on food consumption patterns of indigenous communities of India, who are predominantly smallholder subsistence farmers. Since the last national nutrition survey on indigenous populations of India (NNMB) was conducted more than a decade back, there is a need for a more recent overview on nutritional outcomes among indigenous women of India. Given the fact that indigenous women in India constitute one of the most nutritionally vulnerable groups, it is important to understand their current nutritional profile and explore the factors responsible for poor nutritional outcomes. The present paper thus aims to provide an overview of the nutritional profile of non-pregnant women in reproductive age groups belonging to various indigenous communities in India, by bringing together available information on on (1) their food intake; (2) dietary diversity; (3) their consumption of traditional foods; (4) specific nutrients of concern in their diets; and (5) prevalence of CED and overweight/obesity.

## 2 Materials and Methods

The objective of the paper was to conduct a narrative review on the nutritional outcomes of non-pregnant women belonging to different indigenous communities of India. A narrative approach was chosen over the commonly used systematic approach, since the aim of this review was to describe and synthesize the findings from the published articles and place them to supplement the current lack of knowledge on nutritional scenario among indigenous women. However, we followed a systematic search to avoid subjective selection bias. A literature search was conducted in PubMed and Google Scholar databases, using various combinations of keywords like “nutrition”, “nutritional status”, “nutrient intake”, “dietary intake”, “dietary patterns”, “anthropometry”, “tribal women”, and “India” to identify all studies published between 2010 and 2022. References from retrieved articles were reviewed to identify additional relevant publications. The inclusion criteria were (1) studies conducted on non-pregnant women in the reproductive age group of 15–49 years belonging to a recognized ST as per Article 342 of the Indian constitution [4]; and (2) studies reporting any one of the following outcomes: Food group intake, nutrient intake, traditional food consumption, dietary diversity, and/or BMI classification of indigenous women. The exclusion criteria were studies that did not specify the name of the indigenous communities, as well as review articles of all kinds (narrative and systematic).

The data from selected articles were extracted in MS Excel to record the key variables from each selected study. The data on food group consumption were compared with recommended dietary intakes (RDIs) [21] for moderately active Indian women (based on the principle that indigenous women across India have usually moderate activity levels) to compute the percentage deficit and/or excess in food group intake among indigenous women. In the case of nutrient intakes, the data were compared with estimated average requirements (EARs) for moderately active Indian women [21], wherein the women were categorized as having a very high risk of inadequate intakes when intakes were ≤EAR; or likely a low risk of inadequate intakes when intakes were >EAR. This qualitative classification system has been adapted from a previous study [22], and is useful for estimating the prevalence of nutrient adequacy within the population [22]. The proportion of indigenous women consuming traditional foods and the mean/median dietary diversity scores of indigenous women from different communities were also documented. The estimates of CED and overweight/obesity among women from different indigenous communities were extracted as percentages reported for each category. Women with BMI < 18.5 kg/m^2^ were classified as having CED [23] and women with BMI ≥ 25 were classified as over-weight/obese based on international recommendations [24]. These data were further compared with the NFHS-5 findings [20] on indigenous women of respective states.

## 3 Results and Discussion

**Figure 1:**
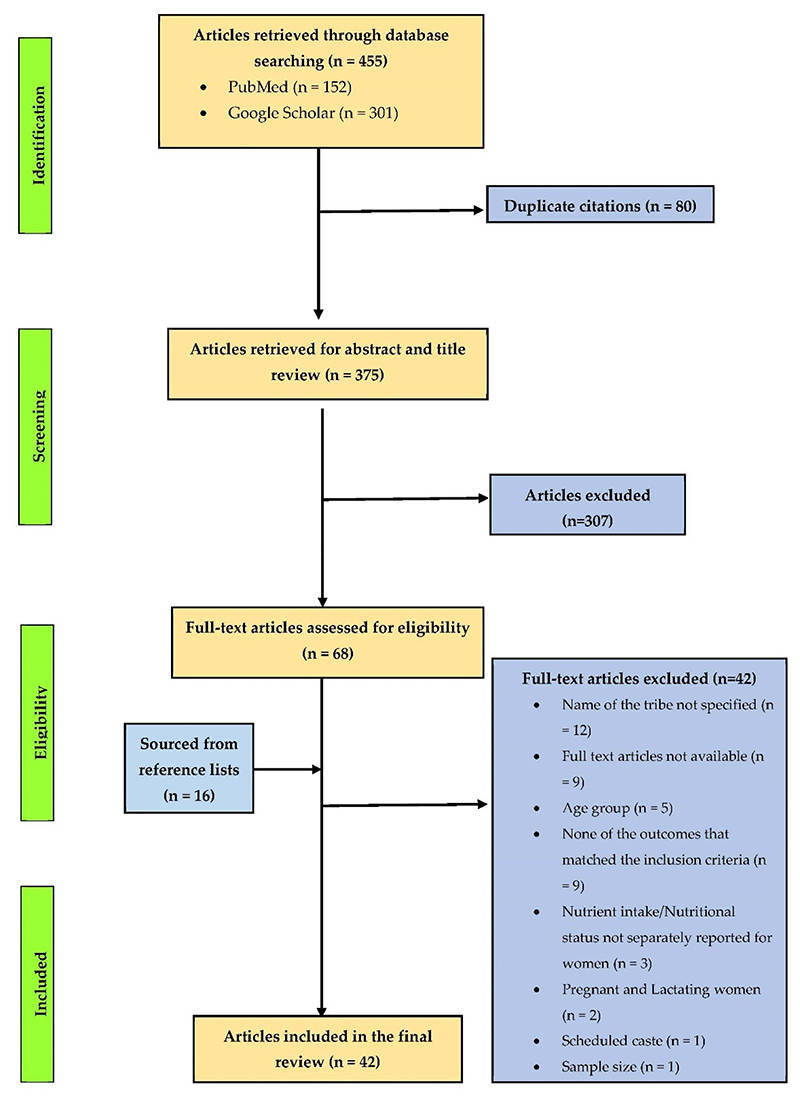
Flow diagram reporting the screening and selection process used in identification of nutritional studies conducted among non-pregnant indigenous women aged 15–49 years in India.

A total of 42 publications that matched the inclusion criteria were included in the final review (Figure 1). An overview of the publications is provided in Supplementary Table S1. The following sections discuss the published literature on various nutritional outcomes in indigenous women belonging to different ethnic communities of India. These findings are further dissected and compared with existing literature to explore the possible factors that influence the diets and nutritional status of indigenous women in India.

### 3.1 Food Group Consumption in Indigenous Women of India

**Figure 2:**
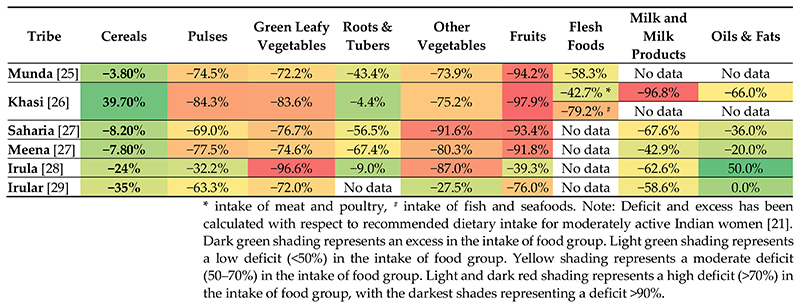
Heat map indicating the percentage deficit/excess in food group consumption among indigenous women of India.

The information on food group intakes were reviewed for indigenous women belonging to six ethnic communities of India: Munda (Jharkhand) [25], Khasi (Meghalaya) [26], Saharia and Meena (Rajasthan) [27], Irula (Kerala) [28] and Irular (Tamil Nadu) [29]. Based on our review, we found exoeptionally low intakes of pulses, GLVs, other vegetables, fruits and flesh foods among indigenous women across all six communities (Supplementary Table S2). In all communities, cereals as well as roots and tubers provided the greatest intakes among the indigenous women, with Khasi women of Meghalaya [26] reporting an excess consumption of cereals in their diet (Figure 2). The relatively better intake of cereals (comprising; mostly rice) among all the six communities may be attributed to its better availability at the household level, as they are usually grown at the farm-level by these communities for household consumption [25,26,30] and can be accessed at subsidized rates through the national food security program—Targeted Public Distribution System (TPDS) [25,30]. This is a concerning issue, as national food security programs and agricultural policies in India have largely focused on ensuring adequate food supply through energy-dense staple crops (such as wheat and rice), which, may fail to address the widespread micronutrient deficiencies among vulnerable sections of the population including women [31–33]. As evident in our review, this approach is leading to a shift in dietary patterns among the vulnerable indigenous communities, by reducing their diets to monotonous, cereal-based meals, with poor consumption of other food groups [31,34]. Reorienting the TPDS to deliver nutrient-dense foods such as various millets and sorghum, could be a potential strategy for improving the diet quality of the vulnerable populations.

Further, in our review, a very high percentage deficit was observed across non-staple food groups like fruits, GLVs and other vegetables; in nearly all six communities (Figure 2). Fruits, in particular, were found to be consumed in the least amounts, with a percentage deficit ranging between if 39% to 98%. Irula women of Tamil Nadu reported the highest deficit in consumption of GLVs (96.6%) [29] while Khasi women reported the highest deficit in consumption of pulses (84.3%) and fruits (97.9%) [26]. These findings are in line with the NNMB tribal survey data (2009), which reported suboptimal intakes (<50% of RDI) of nonstaples like pulses, GLVs, other vegetables in about 50-85% indigenous women across nine states in India [11]. The particularly high deficit in consumption of fruits and vegetables could be attributed to high price inflation and their insufficient domestic production. According to a research on food affordability [35], it was estimated that around 63–76% of the rural Indian population could not afford a recommended diet in 2011, with the cost of a nutritious diet exceeding the expected female wages. Inflation in food prices could, therefore, be a major hindrance towards the affordability of nutritious foods among flee indigenous communities, who are already margiwalised tnd face the generational burden of poverty. Studies have; suggested agricultural diversification as a feasible strategy for reorienting the food systems away from staple grains towards horticulture crops, thereby improving incomes and access to nutrient-dense non-staple foods [35–37].

Although indigenous communities in India are known to consume a predominantly non-vegetarian diet, our review shows a different picture. The daily^1^food consumption data of Khasi women (Meghalaya) [26] shows a diet deficient in meat, poultry and fish (Figure 2), which is in contrast to the wide diversity of ethnic meat based delicacies that are known and consumed by indigenous communities across North-east India [38–42]. The study on Munda women of Jharkhand has also reported a high deficit in flesh food consumption and highlights that the consumption of meat, poultry and fish is mostly limited to 1–2 times a week at the household level [25]. Similar trends have been also reported in Santhal [43], Oraon [44] and Sauria Paharia [30] communities of Jharkhand and Bhil and Bhilala communities of Madhya Pradesh [45]. Consumption of another protein rich source, i.e., the milk and milk products- is also nearly non-existent in all communities, with a percentage deficit of >50% (except in Meena women). It is thus crucial to understand the factors limiting the consumption of animal source foods in indigenous women of India. An article on indigenous communities of Madhya Pradesh [45] has reported that the poor financial conditions of the indigenous populations, along with their overdependence on TPDS, are the two primary reasons for declining meat consumption in indigenous communities across India. Further, the low intake of dairy products in indigenous women could be attributed to the local cultural values of indigenous communities in India, wherein they believe that animal milk is meant for its progeny and is not intended for human use as it is considered a sin [46,47]. In this context, more detailed research on food consumption in indigenous communities would be desirable to understand the current trends and determinants.

### 3.2 Traditional Food Consumption in Indigenous Women of India

**Figure 3:**
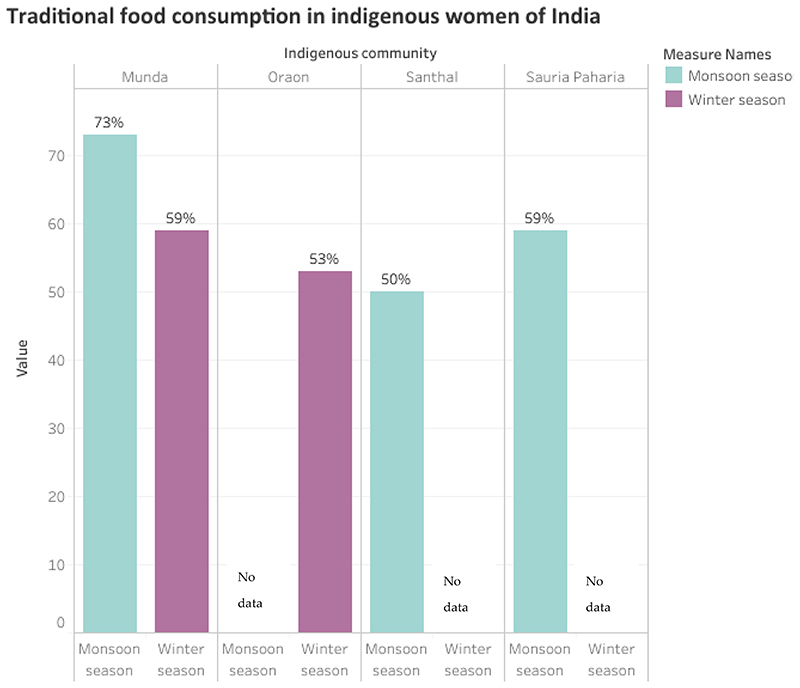
Estimates of traditional food consumption in indigenous women of Jharkhand, India [25,30,44,45].

Traditional foods refer to foods native to a region, that are mainly accessed from natural food environment (i.e., forests, rivers, ponds, lakes, farms, kitchen gardens, wastelands, pastures and roadsides). Considering the globalization of current food systems, traditional foods may also be procured through local markets [48–50]. There are only a few studies that have documented traditional food consumption in indigenous women of India; these include studies conducted on Santhal [43], Oraon [44], Sauria Paharia [30] and Munda women [25] of Jharkhand state of India. Some popular traditional foods consumed by the indigenous communities of Jharkhand include, Red rice, Horse gram (legume), Red amaranth leaves, Koinaar leaves, Basella leaves, Bamboo shoot (vegetable), Kusum (fruit), Eggs of red ants, Snails, among many others [25,30,43,44]. Upon review, we found that the consumption of traditional foods (during the dietary recall period) was reported in >50% of women in all four communities at different time points (monsoon and winter season), with the highest consumption reported in Munda women (73% in monsoon season) (Figure 3). The consumption amounts of traditional foods in Santhal, Oraon and Munda women were found to be quite low in comparison to recommended intakes [21] yet corresponded to significantly higher micronutrient intake in the indigenous women of all the three communities [25,43,44]. Existing literature suggests that indigenous communities in India have access to a variety of wild and cultivated traditional foods that are known to be rich in several nutrients like protein, iron, calcium, zinc, vitamin A, vitamin C, and folate [51], yet their utilization among indigenous communities is on a constant decline [8,9,26,43,44,52]. Different studies have reported several factors contributing to the underutilization of traditional foods in indigenous populations of India- ranging from high opportunity cost of accessing wild foods, limited access to forests and shift towards chemical-intensive agriculture, to changing social values and loss of TEK associated with agro-ecosystems and production practices among younger generations [8,9,53–55]. Additionally, indigenous communities in India are gradually shifting from diverse traditional diets to modern diets, comprising cheap and convenient sources of calories. Qualitative studies conducted with Munda community of Jharkhand [8], Khasi community of Meghalaya [33] and Chakhesang community of Nagaland [33] have revealed that wage labour, increased purchasing power and influence of media and urban lifestyles are leading to an increased dependence on market foods in these communities. However, there is an extensive literature gap on quantitative estimates of ultra-processed food consumption in indigenous communities of India and future research in this domain would be desirable.

The existing TEK in indigenous communities needs to be leveraged, for maximising the consumption of traditional foods. A few studies have reported issues related to deprivation of TEK among indigenous communities, which may be addressed through specific interventions like systematic documentation of local IFs consumed by the tribes, their scientific evaluation in terms of consumption and nutritional content, sustainable utilization and planned advocacy. Additionally, national policies in India may need to devise strategies to mainstream the use of traditional foods in daily diets of indigenous populations. A few state level organizations have taken initiatives to revive indigenous food consumption; for example, Odisha’s FAARM (Food and Agro-Ecological Approaches to Reduce Malnutrition) [56], and Rajasthan’s VAAGDHARA project [57] (supported by NABARD) are working with indigenous communities towards improved access to nutrigardens and wild habitats and use of agro-ecological approach for promoting sustainable food systems. Similar approaches need to be replicated in other states as well, for reversing the shift from traditional foods to modern diets, it indigenous communities. With initiatives lake the National Horticulture Mission (“Mission for Integrated Development of Horticulture (MIDH)”) [58] and POSHAN Atlas [59], efforts may be undertaken to diversify the existing food systems by promoting access to locally sourced wild foods, encouraging agricultural diversification and providing incentives to local farmers to grow nutritionally superior traditional crops [31,60].

### 3.3 Dietary Diversity of Indigenous Women in India

Studies reviewed on dietary diversity of indigenous women in India, reveal the consumption of poor quality diets, with very low individual dietary diversity scores (i.e., minimum diet diversity score for women or MDD-W [61]). In women belonging to Sauria Paharia [30] and Munda communities [25] of Jharkhand, the mean and/or median MDD-W scores were reported to be as low as 3 (ideal score is 5 or more) (Supplementary Table S3). However, even at low dietary diversity scores, a significantly higher intake of both macronutrients and micronutrients was reported in women with MDD-W scores ≥ 3 [25,30]. On the other hand, relatively higher mean MDD-W scores were reported in indigenous women of Meghalaya [62], including Khasi women (mean score of 3.7) and Garo women (mean score of 4.8). It was interesting to note that a majority (60%) of Garo women in Meghalaya reported the consumption of at least 5 food groups during the reference recall period, while only a few (1 or 2) Munda women in Jharkhand reported the consumption of a diverse diet. The findings on Garo women of Meghalaya are consistent with the findings from another study [63] in the same community, that has reported a household dietary diversity score of 5.82, with majority of the households (70%) having a score between 4.41 to 7.23, respectively.

The positive influence of literacy on improved dietary diversity is well documented in the literature, which could be one of the reasons for higher dietary diversity scores in indigenous women of Meghalaya, as their literacy rates were substantially higher (92.6% in Garo and Khasi) in comparison to indigenous women of Jharkhand (50.3% in Sauria Paharia and 64.1% in Munda women) [25,30,62]. Improved socioeconomic household profile was also found to be a significant predictor for dietary diversity in Garo women, while among Munda and Sauria Paharia women, no such association was observed [25,30,62]. Other studies in rural settings of India [36,64] have also identified a significant impact of nutritional awareness on women’s dietary diversity scores, although the evidence in indigenous women is lacking. This could be a crucial point for future interventions as the existing nutrition schemes in India have solely focused on improving nutrition by increasing food availability and/or accessibility without generating nutritional awareness and attempting behavioural change interventions among the beneficiaries.

Majority of the indigenous communities are engaged in subsistence agriculture for their food and livelihood [9,10,26,33,52,62,63,65,66], yet evidence of its impact on women’s diets is limited. In Sauria Paharia and Munda communities of Jharkhand, the Food Accessed Diversity Index (FADI) [25,30], did not show any association with the MDD-W scores, while no such associations were explored in Khasi and Garo women of Meghalaya [62]. A research using production data from rural parts of Bihar, Uttar Pradesh, Odisha, Telangana and Maharashtra, also found a non-significant association between production diversity (including kitchen garden and livestock rearing) and household dietary diversity[67]. These findings highlight that while diversity in food production and access is crucial for improved dietary diversity, there exists other cofounding factors that influence this relationship and hence, need to be explored. Previous studies conducted on Indian rural populations have determined that food markets have a positive and greater impact (than crop diversity) on rural women’s dietary diversity, especially through purchases of pulses/fruits/vegetables [37,64,68,69]. In this context, improving the access of indigenous populations to local markets that offer diverse and affordable food options, could prove to be a viable strategy for improving dietary outcomes.

Another factor that possibly determines dietary diversity among indigenous women is intrahousehold food allocation. An analysis study on Indian rural populations [36] has identified that women in Bihar, Uttar Pradesh and Odisha, consume fewer nutritious food groups (like pulses, GLVs and fruits) than other household members, which was primarily attributed to gender differences in intrahousehold food allocation. A similar scenario is reflected in the NFHS-5 survey findings [20], which reveal that a slightly higher proportion of indigenous men in India reported weekly consumption of fruits (46.5% vs. 37.4%), eggs (57,1% vs. 47.4%) and meat, poultry and fish (58.8% vs. 46%) in comparison to the indigenous women, although the weekly consumption of pulses and GLVs was comparable in both groups. Hence, a more focused and exclusive research on dietary diversity of indigenous communities, at both individual and household levels, would be crucial to understand the gender dynamics of intrahousehold food allocation.

### 3.4 Nutrient Intake of Indigenous Women in India

The nutrient intake data in women was reviewed for 14 indigenous communities of India, which revealed that nearly all communities had reported lower mean/median intakes than EAR for several nutrients (Table 1). Almost all communities (n = 12) had calorie intake lower than EAR, with the lowest calorie intake reported in Sauria Paharia women of Jharkhand [30], followed by Saharia and Meena women of Rajasthan [27]. Protein intakes were found to be slightly higher, with indigenous women in 10 out of 14 communities having a low risk of protein inadequacy (i.e., intake > EAR) (Table 2). These findings are in contrast with the lower intake of pulses, flesh foods and milk and milk products reported in indigenous women from the same communities. As previously discussed in our review, most indigenous communities in India consume a predominantly cereal based diet, which may be contributing to a higher protein intake in these women, resulting in low risk of protein inadequacy. An analysis of protein intakes [70] utilizing the NNMB tribal survey (2009) data revealed that about 70% of the total protein intake in indigenous women was contributed through cereals, while the individual contribution of pulses, flesh foods and milk and milk products was found to be less than 10%., resulting in a PDCAAS score of 76 among indigenous women. Although, a high consumption of cereal based protein is also reported in urban Indian women, contribution from cereals to their total protein intake was lower than 60% with a higher protein contribution from other food groups (especially milk), resulting in a diet with better protein quality [70]. It is, however, important to note that these inferences are drawn from a decade old NNMB survey data and hence, there is a need for more recent comprehensive diet surveys in different sections of the Indian population. Nonetheless, these findings suggest that while indigenous women may be meeting their EAR for protein, the quality of their dietary protein could be a potential cause of concern.

The poor intake of non-staples and low dietary diversity scores in indigenous women, is reflected in their extremely low micronutrient intakes (including vitamin A, folate, iron, calcium and zinc). A high risk of micronutrient inadequacy was reported in indigenous women of Jammu & Kashmir [71,72], Jharkhand [25,30,43,44], Madhya Pradesh [73], Meghalaya [26], Rajasthan [27], Tamil Nadu [28] and Uttar Pradesh [74] (Table 2), especially for vitamin A, iron and calcium. These findings are consistent with the NNMB tribal survey (2009) findings [11], which revealed that majority (80%) of the indigenous women in Tamil Nadu, Odisha and Madhya Pradesh were not able to meet 50% of the RDA for iron, calcium and vitamin A. Results from NNMB rural survey (2012) [75] also highlighted a deficit of 77–80% in vitamin A and folate intake, followed by 47–48% deficit in iron and calcium intake among rural adult women in ten Indian states. The poor nutritional intakes among indigenous women are also reflected in their anaemia status at state levels, particularly in West Bengal (82.3%), Jharkhand (72%), Madhya Pradesh (64.2%), Odisha (71.7%), Rajasthan (61.6%), Uttar Pradesh (51%), Tamil Nadu (59%) and Meghalaya (53.3%) [76], where low intakes of iron, folic acid and vitamin B_12_ were documented. Further, the comparison between NFHS-4 and NFHS-5 data suggest that anaemia prevalence has increased in non-pregnant women across India, with the highest jump reported among indigenous women in West Bengal, Odisha and Jammu and Kashmir [20,76]. Since anaemia is caused by both dietary and non-dietary factors (parasitic infections, gastrointestinal conditions, faulty red-blood cell production), its increasing prevalence in indigenous women is worrisome as it points towards the deteriorating nutritional as well as health conditions of indigenous women across India.

As discussed previously, poverty could be one of the main contributing factors to inadequate micronutrient intake in indigenous communities, as it limits their access to diverse diets, and increases their risk of nutritional insecurity [77,78]. In this context, the functioning of employment schemes like the Mahatma Gandhi National Rural Employment Guarantee Act (MGNREGA) becomes particularly crucial for marginalised indigenous communities. The uptake of MGNREGA has been highest in indigenous communities across India, especially among women, who earn better wages through MGNREGA than market, leading to better economic independence and improved food consumption [79,80]. However, some authors [81,82] have opined that work engagement through MGNREGA leads to ‘feminization of poverty’. A qualitative study [80] conducted in rural areas of Kerala, Tamil Nadu and Odisha, found that women faced a lot of discrimination in work offered through MGNREGA- in terms of lower wages (than men), irregular payment, mistreatment by supervisors, and most importantly, the additional workload (as they are single-handedly burdened with child rearing and domestic responsibilities). A study on rural women of Maharashtra [83] found that women devote equal time as men towards agricultural activities during the peak seasons, but spend a disproportionately higher time in food preparation, domestic work and care activities. The impact of these time trade-offs were found to be significantly associated with a reduced intake of calories, protein, iron, zinc and vitamin A in the women’s diets, with particularly worse nutritional deficits observed among landless women, who resort to working on other people’s farms for additional income [83]. This decrease in nutrient intake was mainly linked to the time constraints faced by the rural women for preparing nutritious meals during the peak agricultural season [83]. Based on these findings, the existing safety net programs may incorporate a more gendered focus to address the disproportionate impacts on women.

### 3.5 Nutritional Status of Indigenous Women in India

**Figure 4:**
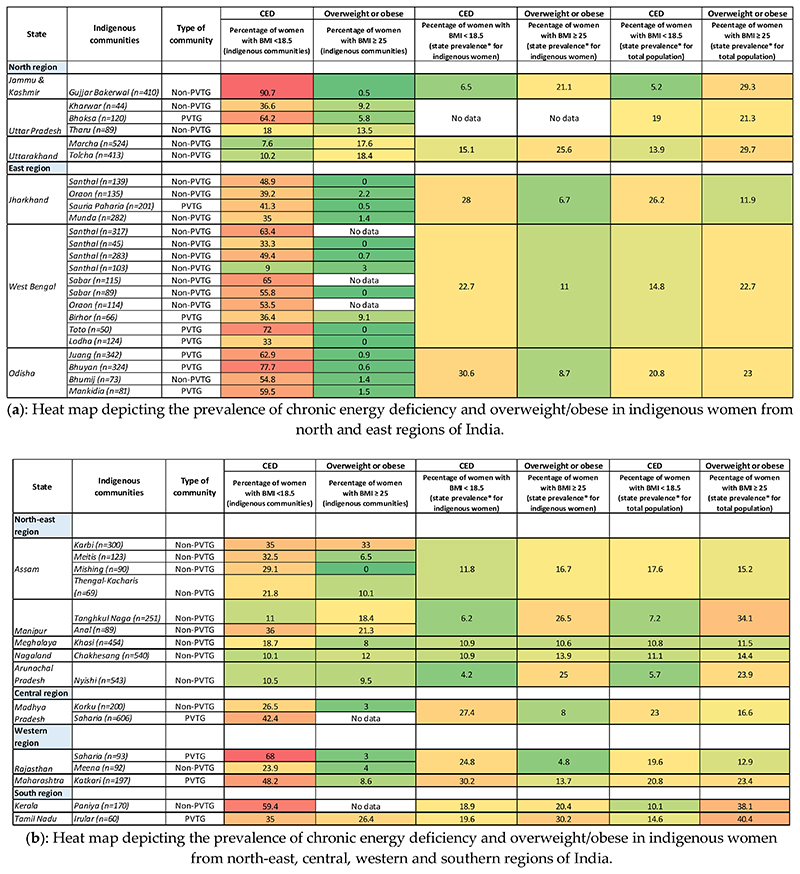
(**a**,**b**) Heat map depicting the prevalence of chronic energy deficiency and overweight/obese in indigenous women from selected states of India. Note: Red shading; indicates a high prevalence (>40%) of chronic energy deficiency or overweight/obese, with darker shades representing a higher prevalence. Orange shading represents a moderate prevalence of 29–39%; Yellow shading represents a prevalence of 16-28%, with green yellowish shades representing a lower prevalence. Green shading represents a low prevalence (<16%), with darker shades representing a lower prevalence. *NFHS-5 (2022) [20].

The data on CED prevalence among indigenous women were available for 36 indigenous communities belonging to sixteen states of India (Figure 4). Upon review, it was found that indigenous women from sixteen ethnic communities of India reported a CED prevalence of >40%, with the highest prevalence (90.7%) reported in Gujjar Bakerwal women of Jammu & Kashmir [71]. Indigenous women, belonging to PVTGs, were particularly observed to have a higher prevalence of undernutrition than other indigenous women. These include, Bhuyan (77%), Juang (62.9%) and Mankirdia (59.5%) women of Odisha [89–91], Toto women (72%) of West Bengal [92], Saharia women (68%) of Rajasthan [27], Bhoksa women (64.2%) of Uttar Pradesh [74], Katkari women of Maharashtra [93] and Sauria Paharia women of Jharkhand [30]. The proportion of CED levels among these women is observed to be far greater than the state averages reported for indigenous women in the recent NFHS-5 (2019-21) [20] survey findings, except in Uttarakhand and Nagaland. Although, it is important to consider that most of these studies used different methodologies for anthropometric measurements and a few studies included in our review were conducted on small sample sizes.

A comparatively lower CED prevalence (<15%) is reported in the studies reviewed on indigenous women from north-eastern region (Manipur, Nagaland and Arunachal Pradesh), which is comparable with the NFHS-5 state-level prevalence on indigenous women. A moderate prevalence of CED is reported in the studies reviewed on indigenous women belonging to Karbi, Mishing, Thengal Kachari and Meitei communities of Assam, and Anal community of Manipur, but this is also accompanied with a moderately high prevalence of overweight and/or obese in these women (Figure 4). The coexistence of over-and undernutrition among indigenous women can also be seen in the NFHS-5 state-level data of Assam, Manipur and Nagaland, which reveal a higher prevalence of overweight and/or obesity in indigenous women, as compared to the total female population in these states. Similar trends are also reported in the NFHS-5 data for Jammu and Kashmir, Uttarakhand, Kerala and Tamil Nadu. The reason for this dual burden of malnutrition in indigenous communities could be attributed to the growing urbanization, which is leading to a shift in occupational patterns, resulting in better household income, reduced physical activity levels, and consequently, a shift from local food consumption towards energy-dense foods among the indigenous communities [94–96]. These rapid changes in diet and lifestyles are contributing to nutrition transition among indigenous populations in India as well as across the globe [97–99].

Many selected studies in this review have also reported better nutritional status among indigenous men in comparison to women, with substantially lower levels of CED (in indigenous communities of Santhals, Sabars and Birhors from West Bengal, Bhuyans, Juangs, Bhumij and Mankidias from Odisha, Meities, Mishing and Thengal Kachari from Assam, Korku from Madhya Pradesh and Kharwar from Uttar Pradesh) [73,89–91,95,100–104,104–106]. Similar results are documented in national survey findings, which report a lower prevalence of CED among indigenous men (19.2%), as opposed to women (25.5%) [76]. The reason for better nutritional status among indigenous men could be attributed to physiological factors [107] and better literacy levels [73,95,100] which coincides with the national figures that report higher literacy rate among indigenous men (68.5%) than women (49.4%) [108]. As previously discussed, gender discrimination could also be one of the possible factors affecting independent access to food by indigenous women [109]. These socio-economic issues are further compounded with heavy work demands, early marriage, childbearing, and rearing, in indigenous women of India, thus increasing their vulnerability to poor nutrition and health outcomes [18,83,92].

In this context, a multi-sectoral approach is highly desirable to address malnutrition in indigenous women of India. Although, India has a plethora of national programs that aim to improve nutrition outcomes by addressing both nutrition-specific and nutrition-sensitive interventions [110], many of these schemes have limited coverage in remote and tribal regions [3,7,43,85,111]. Studies included in our review have cited several operational challenges in the functioning of Integrated Child Development Services (ICDS) and National Health Mission (NHM) in tribal regions, such as non-functional nutrition and health centres, absence of health workers, lack of basic infrastructure, irregular supply of supplementary nutrition to mothers and children, non-transparent reporting system with lack of accountability among many others [43,85,89]. Low access to TPDS was also reported in the indigenous communities of Rajasthan, Meghalaya, Madhya Pradesh and Jharkhand [26,27,43,85]. While some states like Maharashtra (APJ Abdul Kalam Amrut Aahaar Yojana) and Andhra Pradesh (YSR Sampoorna Poshana Plus and Girl Poshana scheme) have taken initiatives by introduction of specific schemes and programmes for indigenous women [112,113], the efforts remain largely fragmented at the national level and lack effective coordination and implementation across different sectors [7,114]. Further, some initiatives by state governments have not considered the cultural eating patterns of the indigenous communities, while planning the interventions. For example, the “Amma Maternity Nutrition Kit Scheme” of Tamil Nadu, provides nutrition kits containing *ayurvedic* (herbal) supplements, ghee, and protein powder, to new mothers; but these items remain underutilised as indigenous populations do not consume ghee, and have no knowledge on incorporation of ayurvedic supplements and protein powder in the diet [55]. The implementation of such schemes reveals a clear disconnect between the planned interventions and the actual support needed by the marginalized populations.

## 4 Limitations

There are some study limitations that must be highlighted. First, our search strategy was limited to PubMed and Google Scholar databases, which may have resulted in the exclusion of any local institutional reports published outside of academia. Second, while our search strategy was based on a wide range of keywords, it may have resulted in the exclusion of relevant studies and research themes. Third, the studies included in our review used different methodologies for data collection, thus making it difficult to compare the findings across different studies. Fourth, most studies included in the review did not capture the seasonality component in dietary data collection, which might have influenced the food and nutrient intakes among indigenous women. Fifth, usual nutrient intakes (probability of consumption on a given day multiplied by the usual intake amount for the day the food is consumed) were not reported in most studies (except in two studies), which is considered ideal for calculating nutrient adequacy at a population level [115]. Sixth, some of the included studies were conducted on a very small sample size, which might not be a true representation of the actual population. Seventh, in our review, we compared the nutrient intake of indigenous women with EAR for a moderately active woman (>18 years), However, there could be a possibility that women from some indigenous communities had heavy or sedentary activity levels, and thus, our interpretation may have over- or underestimated their risk of nutritional inadequacy. Further, the studies included in the review had clubbed the nutrient intake data of women in the age group of 15–49 years, with no segregation in the age-range. This may have impacted the generalizability of the results as the nutritional requirements of adolescent girls in the age range of 15–17 years are different than the requirements prescribed for adult Indian women (>18 years).

## 5 Conclusions

Indigenous women in India contribute substantially to the local economy and share equal responsibilities with men in subsistence activities yet are vulnerable to various forms of malnutrition. In the present review, we selected a total of 42 studies to generate a comprehensive synthesis on food and nutrient intakes, diet quality and anthropometric status among indigenous women from different ethnic communities of India. Our findings showed high percentage deficits in the intake of several food groups, along with poor intake of macronutrients and micronutrients among indigenous women residing in different states of the country. Indices of diet quality in indigenous women were documented in limited studies, which revealed poor dietary diversity as well as low consumption of diverse traditional foods. CED among indigenous women was particularly observed to be a major problem in many ethnic communities across the country, with a dual burden of malnutrition in indigenous women of north-east India. Studies have determined several potential contributing factors toward the poor nutritional outcomes in indigenous women, such as: poverty, high illiteracy in indigenous women, excessive reliance on food grains distributed through TPDS, price inflation of non-staple foods and their insufficient production, gender discrimination in intrahousehold food allocation, high opportunity costs for preparation of nutritious meals, and limited outreach of national food supplementation programs in remotely located regions.

Improving nutrition outcomes for indigenous women thus requires investments to be made in changing the determinants of poor nutrition and health, using a variety of policy instruments and other efforts. The existing higher rates of malnutrition in indigenous communities (particularly in women) than their non-indigenous counterparts may be corrected through leveraging the treasure trove of TEK about the indigenous food systems. In this context, there is a need to acknowledge the significance of Indigenous Peoples’ food systems that make use of various edible and nutritious species of flora and fauna, and hence, may play an important role in enhancing the food and nutritional security of indigenous communities in India. Thus, both targeted (indigenous-specific) and universal (population-wide) policy actions will be essential in improving the nutritional status of indigenous women in India. Targeted approaches must be combined with interventions that improve diet quality, promote agricultural diversification and traditional food consumption, address behaviour change, and focus on strengthening the services and structure of existing nutrition and safety-net programs. Further, despite malnutrition being a persistent problem in indigenous communities over the years, there is no specific Indian policy that focuses on this issue. Therefore, a streamlined indigenous nutrition program, with consideration of local dietary patterns, cultures and livelihoods, could be instrumental in addressing the larger issues of poverty and household food insecurity for improving the overall nutritional outcomes in indigenous populations. POSHAN Abhiyaan, that aims to adopt a holistic approach for improving the functioning of existing services at the grassroots levels-could also be critical in influencing the future nutritional outcomes of indigenous populations. Findings from the present review may thus be crucial for detailed future research on nutritional outcomes in indigenous women, including an in-depth exploration of the disparities faced by them. This is likely to facilitate relevant measures for nurturing the nutritional health of indigenous women in India. The inclusion of indigenous communities in policies and programs and recognition of the various dimensions of their food systems, could be a crucial step towards creating lasting impacts on the nutritional status of indigenous women and children across India.

## Supplementary Material

Table S1

Table S2

Table S3

## Figures and Tables

**Table 1:** Mean nutrient intakes among indigenous women of India.

Tribe	N	EAR ^a^	Energy (kcal/d)	Protein (g/d)	Vitamin A (μg/d)	Vitamin C (mg/d)	Thiamine (mg/d)	Riboflavin (mg/d)	Niacin (mg/d)	Folate (μg/d)	Vitamin B12 (mg/d)	Iron (mg/d)	Calcium (mg/d)	Zinc (mg/d)	Dietary Assessment Method	Random Sample
			2130	36	390	55	1.4	2	12	180	2	15	800	11		
***Jammu & Kashmir***																
Gujjar Bakerwal [71]	410	Mean	1421	40.2	-	-	-	-	-	-	-	7.9	277	-	1	No
		SD	264	7.3								2.3	152.9			
Gujjar [72]	50	Mean	1862	39.4	-	-	-	-	-	-	-	7.8	337.3	-	1	No
		SD	1461	12.8								4.4	292.6			
***Jharkhand***																
Santhal [43]	147	Mean	1969	43.5	923.5	98.9	0.9	0.6	20.9	85.8	0.2	13.9	325.3	7.8	1	Yes
		SD	778	18.9	1364.8	115.9	0.6	0.3	8.7	65.9	0.1	18.9	578.1	3.2		
Oraon [44]	138	Mean	2365	58.3	146.4	61.6	0.7	0.6	27.2	100	0.1	10.1	277.3	9.6	1	Yes
		SD	918	40.3	207.6	46.5	0.3	0.3	13.7	56.4	0.5	8.9	171.03	3.8		
Oraon [84]	100	Mean	2092	41.2	219	25.5	-	-	-	-	-	20.5	288.7	-	1	Yes
		SD	34	4.03	2.03	1.63	-	-	-	-	-	1.4	7.1	-		
Munda [84]	100	Mean	2179	38.9	231.2	27.5	-	-	-	-	-	21.6	305.5	-	1	Yes
		SD	38	4.9	10.2	1.4	-	-	-	-	-	1.1	3.2	-		
Munda [25]	282	Mean	1495	35.6	68.7	48.9	0.5	0.4	7.2	114.2	-	7	120.7	5.6	1	Yes
		SD	269.3	5.3	34.7	9	0.5	0.2	1.5	13.9	-	1.7	7.6	1		
Sauria Paharia [30]	204	Mean	1092	31.6	15.7	12.4	0.3	0.2	5.3	79.4	-	5.2	90.6	4.4	1	Yes
		SD	86.3	3.5	27.6	82.8	0.1	0.1	0.7	22.9	-	1.5	19.4	0.6		
***Madhya Pradesh***																
Saharia [85]	209	Mean	1478	51.7	45 (20, 79) *	6 (2,13) *	1.9	0.7	17.3	55.2	-	20.5	254	-	1	Yes
Korku [73]	602	Mean	1822	37.7	93	16.9	0.7	0.6	9.2	-	-	15.7	170.3	-	2	No
***Meghalaya***																
Khasi [26]	47	Mean	1890	59.2	191.2	33.4	0.7	0.5	13.1	-	-	13.8	322.8	-	1	Yes
		SD	438.3	29.3	279	31.7	0.4	0.2	3.9			10.9	385.2			
***Odisha***																
Desia Khond [86]	80	Mean	2225	33.46	134.5	10.1	0.9	0.6	10.7	47.4	0.15	13.7	195.3	4.8	1	Yes
		SD	262	11.17	228	15.6	0.3	0.53	6.6	15.1	0.42	4.9	56.7	1.3		
***Rajasthan***																
Saharia [27]	93	Mean	1335	42.2	291	23.4	1.6	0.7	13.1	168	0.5	16.1	438.5	6.9	1	No
		SD	287	10.4	281	19.5	0.4	0.2	3.3	54	0.1	4.7	154.3	1.9		
Meena [27]	92	Mean	1386	44.4	442	26.1	1.5	0.8	12.8	166.9	0.2	15.3	531.2	6.7	1	No
		SD	252	8.2	397	18.9	0.1	0.2	2.3	60.3	0.1	3.2	203.5	1.3		
***Tamil Nadu***																
Irula [28]	30	Mean	1830	35.2	558	20	0.4	0.7	8	48	-	12	158	-	2	No
***Uttar Pradesh***																
Bhoksa [74]	120	Mean	1638	42.4	-	-	-	-	-	-	-	13.8	335	-	1	Yes
		SD	243	6.81								2.8	176.4			
***West Bengal***																
Santhal [87]	45	Mean	2180	18	-	-	-	-	-	-	-	-	-	-	3	Yes
		SD	472	2	-	-	-	-	-	-	-	-	-	-		

a —[21]. EAR: Estimated average requirements, RDA: Recommended dietary allowances, SD: Standard deviation, * median and IQR presented. 1—24 h recall. 2—Food weighment method. 3—Quantitative food frequency questionnaire [88].

**Table 2:** Risk of nutritional inadequacy among indigenous women of India.

Tribe	Energy	Protein	Vitamin A	Vitamin C	Thiamine	Riboflavin	Niacin	Folate	Vitamin B12	Iron	Calcium	Zinc
**Jammu & Kashmir**												
Gujjar Bakerwal [71]	High risk	Low risk	No data	No data	No data	No data	No data	No data	No data	High risk	High risk	No data
Gujjar [72]	High risk	Low risk	No data	No data	No data	No data	No data	No data	No data	High risk	High risk	No data
**Jharkhand**												
Santhal [43]	High risk	Low risk	Low risk	Low risk	High risk	High risk	Low risk	High risk	High risk	High risk	High risk	High risk
Oraon [44]	Low risk	Low risk	High risk	Low risk	High risk	High risk	Low risk	High risk	High risk	High risk	High risk	High risk
Oraon [84]	High risk	Low risk	High risk	High risk	No data	No data	No data	No data	No data	Low risk	High risk	No data
Munda [84]	Low risk	Low risk	High risk	High risk	No data	No data	No data	No data	No data	Low risk	High risk	No data
Munda [25]	High risk	High risk	High risk	High risk	High risk	High risk	High risk	High risk		High risk	High risk	High risk
Sauria Paharia [30]	High risk	High risk	High risk	High risk	High risk	High risk	High risk	High risk		High risk	High risk	High risk
**Madhya Pradesh**												
Saharia [85]	High risk	Low risk	High risk	High risk	Low risk	High risk	Low risk	High risk	No data	Low risk	High risk	No data
Korku [73]	High risk	Low risk	High risk	High risk	High risk	High risk	High risk	No data	No data	Low risk	High risk	No data
**Meghalaya**												
Khasi [26]	High risk	Low risk	High risk	High risk	High risk	High risk	Low risk	No data	No data	High risk	High risk	No data
**Odisha**												
Desia Khond [86]	Low risk	High risk	High risk	High risk	High risk	High risk	High risk	High risk	High risk	High risk	High risk	High risk
**Rajasthan**												
Saharia [27]	High risk	Low risk	High risk	High risk	Low risk	High risk	Low risk	High risk	High risk	Low risk	High risk	High risk
Meena [27]	High risk	Low risk	Low risk	High risk	Low risk	High risk	Low risk	High risk	High risk	Low risk	High risk	High risk
**Tamil Nadu**												
Irula [28]	High risk	High risk	Low risk	High risk	High risk	High risk	High risk	High risk	No data	High risk	High risk	High risk
**Uttar Pradesh**												
Bhoksa [74]	High risk	Low risk	No data	No data	No data	No data	No data	No data	No data	High risk	High risk	High risk
**West Bengal**												
Santhal [87]	Low risk	High risk	No data	No data	No data	No data	No data	No data	No data	No data	No data	No data

## Data Availability

No new data were created or analyzed in this study. Data sharing is not applicable to this article.
