# Supplementary material for: Diet Quality, Nutritional Adequacy and Anthropometric Status among Indigenous Women of Reproductive Age Group (15–49 Years) in India: A Narrative Review
Source: Dietetics (Basel). Author manuscript; Available in PMC 2023 Aug 25. (PMC7614979; doi:10.3390/dietetics2010001)
Supplement: Table S1 [file EMS182764-supplement-Table_S1.pdf]

Table S1. List of studies included in review.

S No.	Study settings	Tribal group/ Sample size of women in reproductive age group	Category of tribal group	Method for data collection	Random sampling (Yes/No)	Outcomes					Reference
						Food group intake of women	Nutrient intake of women	Anthropo-metric status	Traditional food consumption	Diet diversity scores	
1.	Bankar district, West Bengal	Sabar (n=115)	Non-PVTG	• Anthropometric measurements of height and weight were taken using standard techniques [1]<br>• Nutritional status evaluated using internationally accepted guidelines [2]	No			✓			[3]
2.	Papum Pare district, Arunachal Pradesh	Nyishi (n=543)	Non-PVTG	• Anthropometric measurements of height and weight were taken using standard techniques [4]<br>• Coefficient of reliability tested using TEM.<br>• Nutritional status evaluated using internationally accepted guidelines [5]	No			✓			[6]
3.	Cachar district, Assam	Meitis (n=141)	Non-PVTG	• Anthropometric measurements of height and weight were taken using standard techniques [7]<br>• Nutritional status evaluated using internationally accepted guidelines [2]	No			✓			[8]
4.	Alipurduar district, West Bengal	Oraon (n=114)	Non-PVTG	• Anthropometric measurements of height and weight were taken using standard techniques [7]<br>• Nutritional status evaluated using internationally accepted guidelines [5]	No			✓			[9]
5.	Baran district, Rajasthan	Saharia (n=100) and Meena (n=100)	Saharia: PVTG; Meena: Non-PVTG	• Anthropometric measurements of height and weight were taken using standard techniques (no reference cited)<br>• 24-hr recall method for two non-consecutive days was used to assess nutrient intake<br>• Nutrient intake data compared with RDA/RDI for moderately active adult Indian woman [10,11]	No	✓	✓	✓			[12]

S No.	Study settings	Tribal group/ Sample size of women in reproductive age group	Category of tribal group	Method for data collection	Random sampling (Yes/No)	Outcomes					Reference
						Food group intake of women	Nutrient intake of women	Anthropo-metric status	Traditional food consumptio n	Diet diversity scores	
6.	West Khasi Hills district, Meghalaya	Khasi (n=454 for anthropometr y and n=47 for dietary assessment)	Non-PVTG	• Anthropometric measurements of height and weight were taken using standard techniques [13]<br>• A 1-day 24-hr recall method was conducted for every sixth household [14]<br>• Nutrient intake data compared with RDA and RDI for a moderately active adult Indian woman [10,11]	Yes	✓	✓	✓			[15]
7.	Betul district, Madhya Pradesh	Korku (n=903)	Non-PVTG	• Raw food weighment method was used for detailed study of food intake of samples<br>• Nutrient intake data compared with RDA/RDI for a moderately active adult Indian woman [11]	Yes		✓				[16]
8.	Purulia district,<br><br>West Bengal	Santal (n=317)	Non-PVTG	• Anthropometric measurements of height and weight were taken using standard techniques [1]<br>• Nutritional status evaluated using internationally accepted guidelines [2]	No			✓			[17]
9.	Purulia district,<br><br>West Bengal	Birhor (n=75)	PVTG	• Anthropometric measurements of height and weight were taken using standard techniques [1]<br>• Nutritional status evaluated using internationally accepted guidelines [2]	No			✓			[18]
10.	Purba Medinipur district,<br><br>West Bengal	Santhal (n=45)	Non-PVTG	• Anthropometric measurements of height and weight were taken using standard techniques[7]<br>• Nutritional status evaluated using internationally accepted guidelines [19]<br>• Diet history questionnaire with defined questions on frequency and portion size was used [20]	Yes		✓	✓			[21]

S No.	Study settings	Tribal group/ Sample size of women in reproductive age group	Category of tribal group	Method for data collection	Random sampling (Yes/No)	Outcomes					Reference
						Food group intake of women	Nutrient intake of women	Anthropo-metric status	Traditional food consumption	Diet diversity scores	
11.	Pashcim Medinipur district,<br>West Bengal	Santhal (n-103) and Lodha (n=124)	Santhal: Non-PVTG; Lodha: PVTG	• Anthropometric measurements of height and weight were taken using standard techniques [1]<br>• Nutritional status evaluated using internationally accepted guidelines [2]	No			✓			[22]
12.	Raigad district,<br>Maharashtra	Katkari (n=219)	PVTG	• Anthropometric measurements of height and weight were taken using standard techniques and equipments<br>• Nutritional status evaluated using internationally accepted guidelines [2]	No			✓			[23]
13.	Kandhamal district,<br>Odisha	Desia Khonds (n=80)	Non-PVTG	• 24-hour recall method used for dietary intake assessment.<br>• Details on nutrient intake analysis not mentioned.	Yes		✓				[24]
14.	Bankura district,<br>West Bengal	Sabar (n=115)	Non-PVTG	• Anthropometric measurements of height and weight were taken using standard techniques [1]<br>• TEM were computed and found within acceptable limits [4]<br>• Nutritional status evaluated using internationally accepted guidelines [2]	No			✓			[25]
15.	Sheopur, Shivpuri and Guna district, Madhya Pradesh	Saharia<br>(n=606)	PVTG	• Anthropometric measurements of height and weight were taken using standard techniques [13,19]<br>• 1 day 24-hour recall method was used to assess dietary intake<br>• Nutrient intake data compared with RDA/RDI for moderately active adult Indian woman [11]<br>• Nutritional status evaluated using internationally accepted guidelines [2]	Yes		✓	✓			[26]

S No.	Study settings	Tribal group/ Sample size of women in reproductive age group	Category of tribal group	Method for data collection	Random sampling (Yes/No)	Outcomes					Reference
						Food group intake of women	Nutrient intake of women	Anthropo-metric status	Traditional food consumption	Diet diversity scores	
16.	Godda district, Jharkhand	Santhals (n=139)	Non-PVTG	• Anthropometric measurements of height and weight were taken using standard protocols and equipments (weighing balance and anthropometer rod)<br>• A two-day 24-hour recall method was used to assess dietary intake<br>• Household dietary patterns assessed using pretested FFQ<br>• Nutrient intake data compared with RDA/RDI for moderately active adult Indian woman [11]<br>• Nutritional status was evaluated using internationally accepted guidelines [5]	Yes		✓	✓	✓		[27]
17.	Gumla district, Jharkhand	Oraon (n=135)	Non-PVTG	• Anthropometric measurements of height and weight were taken using standard protocols and equipments (weighing balance and anthropometer rod)<br>• A two-day 24 h DR was taken for one woman of reproductive age per HH<br>• Household dietary patterns assessed using pretested FFQ<br>• Nutrient intake data compared with RDA/RDI for moderately active adult Indian woman [11]<br>• Nutritional status was evaluated using internationally accepted guidelines [5]	Yes		✓	✓	✓		[28]
18.	Baleswar district, Odisha	Bhumij (n=73)	Non-PVTG	• Anthropometric measurements of height and weight were taken using standard techniques (no reference).<br>• Nutritional status was evaluated using internationally accepted guidelines [2]	No			✓			[29]
19.	Keonjhar & Angul districts, Odisha	Bhuyan (n=324)	PVTG	• Anthropometric measurements of height and weight were taken using standard techniques [5]<br>• TEM were computed and were found within acceptable limits [4]<br>• Nutritional status evaluated using internationally accepted guidelines [2]	No			✓			[30]

S No.	Study settings	Tribal group/ Sample size of women in reproductive age group	Category of tribal group	Method for data collection	Random sampling (Yes/No)	Outcomes					Reference
						Food group intake of women	Nutrient intake of women	Anthropo-metric status	Traditional food consumptio n	Diet diversity scores	
20.	Keonjhar district, Odisha	Juangs<br>(n=423)	PVTG	• Anthropometric measurements of height and weight were taken using standard techniques [31]<br>• TEM were computed and were found within acceptable limits [4]<br>• Nutritional status evaluated using internationally accepted guidelines [2]	No			✓			[32]
21.	Mayurbhanj district, Odisha	Mankdias<br>(n=136)	PVTG	• The anthropometric data (height and weight) was collected using the standard techniques [1]<br>• TEM were computed and were found within acceptable limits [33]<br>• Nutritional status was evaluated using internationally accepted guidelines [2]	No			✓			[34]
22.	Kamrup district, Assam	Karbi<br>(n=300)	Non-PVTG	• The anthropometric data (height and weight) was collected using the standard techniques [1]<br>• Nutritional status was evaluated using internationally accepted guidelines [5]	Yes			✓			[35]
23.	Anantnag, Baramulla, Gandarbal and Srinagar districts, Jammu & Kashmir	Gujjar Bakerwal<br>(n=410)	Non-PVTG	• Anthropometric measurements of height and weight were taken using standard techniques<br>• Nutritional status was evaluated using internationally accepted guidelines [5]<br>• 24-hour recall method was used to assess dietary intake.<br>• Details on nutrient intake analysis are not mentioned.	No		✓	✓			[36]
24.	Mirzapur district, Uttar Pradesh	Kharwar<br>(n=76)	Non-PVTG	• Anthropometric measurements of height and weight were taken using standard techniques [7]<br>• Nutritional status was evaluated using internationally accepted guidelines [5]	Yes			✓			[37]

S No.	Study settings	Tribal group/ Sample size of women in reproductive age group	Category of tribal group	Method for data collection	Random sampling (Yes/No)	Outcomes					Reference
						Food group intake of women	Nutrient intake of women	Anthropo-metric status	Traditional food consumptio n	Diet diversity scores	
25.	Chandel district, Manipur	Anal Naga (n=89)	Non-PVTG	• Anthropometric measurements of height and weight were taken using standard techniques Nutritional status assessed using internationally accepted guidelines [2]	Yes			✓			[38]
26.	Bijnor district, Uttar Pradesh	Bhoksa (n=120)	PVTG	• Anthropometric measurements of height and weight were taken using standard techniques.<br>• 24-hour recall method was used to assess dietary intake. Details on nutrient intake analysis not mentioned.<br>• Nutritional status assessed using internationally accepted guidelines [2]	Yes		✓	✓			[39]
27.	Phek district, Nagaland	Chakhesang (n=540)	Non-PVTG	• Anthropometric measurements of height and weight were taken and standardized within accepted margins of error [40]<br>• Details on nutritional status assessment not mentioned	Yes			✓			[41]
28.	Chamoli district, Uttarakhand	Bhotia (Marcha and Tolcha) (n=937)	Non-PVTG	• The anthropometric measurements of height and weight was collected using the standard techniques [7]<br>• Nutritional status was assessed using BMI cut-off points for Asians [5]	No			✓			[42]
29.	Wayanad district, Kerala	Paniyar, Kurichyar, Kattunaikkar (n=193)	Paniyar, Kurichyar: Non-PVTG, Kattunaik kar: PVTG	• Anthropometric measurements of height and weight were taken using standard techniques (no reference mentioned).<br>• Nutritional status was assessed using BMI cut-off points for Asians [5]	Yes			✓			[43]
30.	Udham Singh Nagar district,	Tharu (n=176)	Non-PVTG	• The anthropometric data (height and weight) was collected using the standard techniques [1]	Yes			✓			[44]

S No.	Study settings	Tribal group/ Sample size of women in reproductive age group	Category of tribal group	Method for data collection	Random sampling (Yes/No)	Outcomes					Reference
						Food group intake of women	Nutrient intake of women	Anthropo-metric status	Traditional food consumptio n	Diet diversity scores	
	Uttarakhand			• Nutritional status was evaluated using internationally accepted guidelines [2]							
31.	Ukhrul district, Manipur	Tangkhul Naga<br>(n=346)	Non-PVTG	• Anthropometric measurements of height and weight were taken using standard techniques [7]<br>• Nutritional status was evaluated using BMI cut-off points for Asians [5]	No			✓			[45]
32.	Coimbatore, Kerala	Irula<br>(n=100)	Non-PVTG	• Weighment method for three days was conducted for dietary intake assessment, and compared with RDA for moderately active Indian women [11]<br>• Structured interview schedule used to assess household dietary patterns<br>• Height and weight were measured. No reference was given for techniques and assessment	No		✓				[46]
33.	Betul district,<br>Madhya Pradesh	Korku<br>(n=200)	Non-PVTG	• Anthropometric measurements of height and weight were taken using standard techniques<br>• Details on nutritional status assessment not mentioned.	No			✓			[47]
34.	Lakhimpur district, Assam	Mishing (n=136), Thengal Kacharis (n=116)	Non-PVTG	• Anthropometric measurements such as height and weight were taken using standard protocols (no reference given)<br>• Nutritional status was evaluated using internationally accepted guidelines [48]	No			✓			[49]
35.	Jalpaiguri district,<br>West Bengal	Toto<br>(n=50)	PVTG	• Anthropometric measurements such as height and weight were taken using standard protocols [50]<br>• Nutritional status was evaluated using internationally accepted guidelines [51]	Yes			✓			[52]

S No.	Study settings	Tribal group/ Sample size of women in reproductive age group	Category of tribal group	Method for data collection	Random sampling (Yes/No)	Outcomes					Reference
						Food group intake of women	Nutrient intake of women	Anthropo-metric status	Traditional food consumption	Diet diversity scores	
36.	Birbhum district, West Bengal	Santhal (n=288)	Non-PVTG	• Anthropometric measurements such as height and weight were taken using standard protocols.<br>• Nutritional status was evaluated using internationally accepted guidelines [53,54]	Yes			✓			[55]
37.	Kancheepuram district, Tamil Nadu	Irular (n=60)	PVTG	• Anthropometric measurements such as height and weight were taken using standard protocols 24-hour recall method was used to assess dietary intake.<br>• Nutritional status was evaluated using internationally accepted guidelines [2]	No	✓		✓			[56]
38.	Gumla district, Jharkhand	Oraon and Munda (n=200)	Non-PVTG	• 24-hour recall method for 3 consecutive days was used for dietary intake assessment.<br>• Details on nutrient intake analysis not mentioned	Yes		✓				[57]
39.	Bandipora State: Jammu & Kashmir	Gujjar (n=50)	Non-PVTG	• Tool for nutrient intake assessment not mentioned	No		✓				[58]
40.	Godda district, Jharkhand	Sauria Paharia (n=204 for dietary assessments; n=201 for anthropometric assessments)	PVTG	• 24-hour recall method for 2 non-consecutive days was used for dietary intake assessment.<br>• Household dietary patterns assessed using pretested FFQ<br>• Nutrient intake data compared with RDA and EAR for moderately active adult Indian woman [11]<br>• The diet diversity scores were calculated by adding the number of food groups (for food items with amounts ≥15 g) consumed by the women as reported in the 24 HDR. Women who consumed ≥5 food groups were considered to be meeting recommendations for minimum dietary diversity [59].	Yes		✓	✓	✓	✓	[60]

S No.	Study settings	Tribal group/ Sample size of women in reproductive age group	Category of tribal group	Method for data collection	Random sampling (Yes/No)	Outcomes					Reference
						Food group intake of women	Nutrient intake of women	Anthropo-metric status	Traditional food consumption	Diet diversity scores	
				• Anthropometric measurements such as height and weight were taken using standard protocols and equipments (infantometer, stadiometer and digital weighing balance)							
41.	Khunti district, Jharkhand	Munda (n=282)	Non-PVTG	• 24-hour recall method for 2 non-consecutive days was used for dietary intake assessment.<br>• Household dietary patterns assessed using pretested FFQ<br>• Nutrient intake data compared with RDA and EAR for moderately active adult Indian woman [11]<br>• The diet diversity scores were calculated by adding the number of food groups (for food items with amounts ≥15 g) consumed by the women as reported in the 24 HDR. Women who consumed ≥5 food groups were considered to be meeting recommendations for minimum dietary diversity [61].<br>• Anthropometric measurements such as height and weight were taken using standard protocols and equipments (infantometer, stadiometer and digital weighing balance)	Yes	✓	✓	✓	✓	✓	[62]
42.	East Khasi Hills, West Khasi Hills, West Janitia Hills, West Garo Hills, Ri Bhoi district, Meghalaya	Khasi and Garo (n=276)	Non-PVTG	• 24-hour recall was used to capture detailed information about all foods and beverages consumed by a woman in the past 24 hours<br>• The data of 24-hour recall was used to calculate the dietary diversity using a simple count of food groups that the women of reproductive age had consumed over the past 24 hours. Food consumed was then categorized according to the 10 food groups given in the Minimum Dietary Diversity for Women: A Guide to Measurement [61].	Yes					✓	[63]

RDA-Recommended dietary allowances; RDI-Recommended dietary intake; EAR-Estimated average requirements; TEM-Technical errors of measurement; FFQ-Food Frequency Questionnaire

References

1. Lohman, T.G.; Roche, A.F.; Martorell, R. *Anthropometric Standardization Reference Manual*; Human Kinetics Books: Champaign, IL, USA, 1988; ISBN 978-0-87322-121-4.
2. WHO Expert Committee on Physical Status: The Use and Interpretation of Anthropometry (1993: Geneva, S.; Organization, W.H. *Physical Status: The Use of and Interpretation of Anthropometry, Report of a WHO Expert Committee*; World Health Organization, 1995; ISBN 978-92-4-120854-3.
3. Bhandari, S.; Ghosh, M.; Bose, K. Socio-Demographic Characteristics and Prevelance of Undernutrition among Adult Sabars of Bankura District, West Bengal, India. *IJALSR* **2019**, *2*, 1–10, doi:10.31632/ijalsr.2019v02i01.001.
4. Hall, J.; Allanson, J.; Gripp, K.; Slavotinek, A. *Handbook of Physical Measurements*; 2 edition; Oxford University Press: Oxford; New York, USA, 2006; ISBN 978-0-19-530149-6.
5. WHO Expert Consultation Appropriate Body-Mass Index for Asian Populations and Its Implications for Policy and Intervention Strategies. *Lancet* **2004**, *363*, 157–163, doi:10.1016/S0140-6736(03)15268-3.
6. Bharali, N.; Mondal, N.; Singh, K.N. Prevalence of Undernutrition, Overweight and Obesity among Nyishi Tribal Women of Arunachal Pradesh, Northeast India. *Human Biology Review* **2017**, *6*, 63–78.
7. Weiner, J.S.; Lourie, J.A. *Practical Human Biology*; Academic Press: London; New York, 1981; ISBN 978-0-12-741960-2.
8. Barbhuïya, A.F.G.İ.; Das, R. Gender Differences in Nutritional Status among the Adult Meiteis of Cachar District of Assam, India. *Eurasian Journal of Anthropology* **2014**, *4*, 36–44.
9. Bhattacharya, A.; Mukherjee, S.; Roy, S.K. Nutritional Assessment of Oraons of West Bengal: A Comparison between Biochemical and Anthropometric Methods. *Anthropological Review* **2019**, *82*, 297–311, doi:10.2478/anre-2019-0022.
10. NIN *Dietary Guidelines for Indians- A Manual*; National Institute of Nutrition, Indian Council of Medical Research, 2011;
11. ICMR Nutrient Requirements & Recommended Dietary Allowances for Indians (1990, Reprinted 2008) 2nd Edition - 2010) By B.S. Narasinga Rao, B. Sivakumar) | Indian Council of Medical Research | Government of India Available online: https://www.icmr.nic.in/content/nutrient-requirements-recommended-dietary-allowances-indians-1990-reprinted-2008-2nd-edition (accessed on 28 September 2019).
12. Chishty, S.; Singh, N. Nutritional Status of Tribal Women (Saharia and Meena), Baran District of Rajasthan, India. *Nutrition & Food Science* **2018**, *48*, doi:10.1108/NFS-01-2018-0018.

13. Jelliffe, D.B.; Jelliffe, E.F.P. *Community Nutritional Assessment: With Special Reference to Less Technically Developed Countries*; Oxford University Press: Oxford, New York, USA, 1990; ISBN 978-0-19-261218-2.
14. Thimmayamma, B.V.S.; Rao, D.H. A Comparative Study of the Oral Questionnaire Method with Actual Observation of the Dietary Intake of Pre-School Children. *Indian Journal of Nutrition and Dietetics* **1969**, *6*, 177–181.
15. Chyne, D.A.L.; Meshram, I.I.; Rajendran, A.; Kodali, V.; Getti, N.; Roy, P.; Kuhnlein, H.V.; Longvah, T. Nutritional Status, Food Insecurity, and Biodiversity among the Khasi in Meghalaya, North-East India. *Matern Child Nutr* **2017**, *13 Suppl 3*, doi:10.1111/mcn.12557.
16. Das, M. Study of Nutritional Status of Korku Tribes in Betul District of Madhya Pradesh. *Studies of Tribes and Tribals* **2010**, *8*, 31–36, doi:10.1080/0972639X.2010.11886611.
17. Das, S.; Bose, K. Body Mass Index and Chronic Energy Deficiency among Adult Santals of Purulia District, West Bengal, India. *Journal of Human Sciences* **2010**, *7*, 488–503.
18. Das, S.; Mahata, M. Nutritional Profile of Adult Birhors of Purulia: A Particularly Vulnerable Tribal Group of, West Bengal, India. *Asian Academic Research Journal of Multidisciplinary;* **2013**, *1*, 262–276.
19. Gibson, R.S. An Interactive 24-Hour Recall for Assessing the Adequacy of Iron and Zinc Intakes in Developing Countries. **2008**, 160.
20. Hebert, J.R.; Gupta, P.C.; Bhonsle, R.B.; Murti, P.R.; Mehta, H.; Verghese, F.; Aghi, M.; Krishnaswamy, K.; Mehta, F.S. Development and Testing of a Quantitative Food Frequency Questionnaire for Use in Kerala, India. *Public Health Nutr* **1998**, *1*, 123–130, doi:10.1079/phn19980019.
21. Dash, K.; Adhikari, A. Comparison of Nutritional Status between Santal Tribal Women and Bengali Women of Purba Medinipur, West Bengal. *Indian Journal of Biological Sciences* **2017**, *23*, 24–33.
22. De, R.; Kundu, J. Nutritional Status of Adult Female Tribes in Different Villages of Jhargram, West Bengal. *WORLD JOURNAL OF PHARMACY AND PHARMACEUTICAL SCIENCES* **2016**, *5*, doi:10.20959/wjpps201612-8258.
23. Deo, M.G.; Pawar, P.V.; Kanetkar, S.R.; Kakade, S.V. Prevalence and Risk Factors of Hypertension and Diabetes in the Katkari Tribe of Coastal Maharashtra. *J Postgrad Med* **2017**, *63*, 106–113, doi:10.4103/0022-3859.194204.
24. Garnaik, D.; Devadarshini, C.; Jena, D. Nutritional Status of Tribal Women of Kandhamal District, Odisha. *The Pharma Innovation Journal* **2019**, *8*, 105–107.

25. Ghosh, M.; Bhandari, S.; Bose, K. Anthropometric Characteristics and Nutritional Status of Adult Sabars of Bankura District, West Bengal. *Human Biology Review* **2018**, *7*, 13.
26. Ghosh-Jerath, S.; Singh, A.; Bhattacharya, A.; Ray, S.; Yunus, S.; Zodpey, S.P. Dimensions of Nutritional Vulnerability: Assessment of Women and Children in Sahariya Tribal Community of Madhya Pradesh in India. *Indian Journal of Public Health* **2013**, *57*, 260, doi:10.4103/0019-557X.123268.
27. Ghosh-Jerath, S.; Singh, A.; Magsumbol, M.S.; Lyngdoh, T.; Kamboj, P.; Goldberg, G. Contribution of Indigenous Foods towards Nutrient Intakes and Nutritional Status of Women in the Santhal Tribal Community of Jharkhand, India. *Public Health Nutr* **2016**, *19*, 2256–2267, doi:10.1017/S1368980016000318.
28. Ghosh-Jerath, S.; Singh, A.; Lyngdoh, T.; Magsumbol, M.S.; Kamboj, P.; Goldberg, G. Estimates of Indigenous Food Consumption and Their Contribution to Nutrient Intake in Oraon Tribal Women of Jharkhand, India. *Food Nutr Bull* **2018**, *39*, 581–594, doi:10.1177/0379572118805652.
29. Goswami, M.; Dash, B.; Dash, N.C. Nutritional Status of the Bhumija of Baleswar, Orissa. *Indian Anthropologist* **2010**, *40*, 55–70, doi:10.2307/41920110.
30. Goswami, M. BODY MASS INDEX AND CHRONIC ENERGY DEFICIENCY OF ADULT BHUYANS: A VULNARABLE TRIBAL GROUP OF KEONJHAR AND ANUGUL DISTRICTS OF ODISHA, INDIA. *Indian journal of physical anthropology and human genetics* **2012**, *31*, 39–47.
31. Lee, R.D.; Nieman, D.C. *Nutritional Assessment*; 4th edition; McGraw Hill Higher Education: Boston, 2006; ISBN 978-0-07-125426-7.
32. Goswami, M. Prevalence of Under-Nutrition among the Juangs A Study on a Particularly Vulnerable Tribal Group of Odisha, India. **2013**, *9*, 61–66.
33. Ulijaszek, S.J.; Kerr, D.A. Anthropometric Measurement Error and the Assessment of Nutritional Status. *British Journal of Nutrition* **1999**, *82*, 165–177, doi:10.1017/S0007114599001348.
34. Goswami, M. Anthropometric Characteristics and Chronic Energy Deficiency of the Mankidias - A PTG of Northern Odisha, India. *Studies of Tribes and Tribals* **2015**, *13*, 88–93, doi:10.1080/0972639X.2015.11886715.
35. Goswami, R.; Bhattacharya, M. Nutritional Status among Adult Karbi Women of Kamrup District, Assam. *International Research Journal of Social Sciences* **2015**, *4*, 17–20.

36. Hamid, T.; Vaida, N. A Study on Nutritional Status of Scheduled Tribe (Gujjar and Bakerwal) Women of Kashmir. *International Journal of Home Science* **2017**, *3*, 203–205.
37. Jaiswal, A. A Study on Body Mass Index and Prevalence of Chronic Energy Deficiency among Adult Kharwar Tribes of India. *Global Journal of Anthropology Research* **2015**, *2*, 50–55, doi:10.15379/2410-2806.2015.02.01.05.
38. Khiloni, I. A STUDY ON THE IMPACT OF MATERNAL STATUS ON CHILD NUTRITION AMONGST THE ANAL NAGA TRIBE OF CHANDEL DISTRICT, MANIPUR. *International Journal of Research and Analytical Reviews* **2018**, 555–559.
39. Lamba, A.; Garg, V. Nutrition Profile of the Tribal (Bhoksa) Women in Bijnor District, Uttar Pradesh. **2018**, *7*, 82–84.
40. Jp, H. Standardization of Quantitative Epidemiological Methods in the Field. *Bol Oficina Sanit Panam* **1974**, *76*, 375–384.
41. Longvah, T.; Khutsoh, B.; Meshram, I.I.; Krishna, S.; Kodali, V.; Roy, P.; Kuhnlein, H.V. Mother and Child Nutrition among the Chakhesang Tribe in the State of Nagaland, North-East India. *Matern Child Nutr* **2017**, *13 Suppl 3*, doi:10.1111/mcn.12558.
42. Mandal, C.; Adak, D.; Biswas, S.; Bharati, P. A Study on BMI among the Bhotia of Uttaranchal, India. *Asian Pacific Journal of Tropical Disease* **2011**, *1*, 55–58, doi:10.1016/S2222-1808(11)60014-5.
43. Mohandas, S.; Amritesh, K.; Lais, H.; Vasudevan, S.; Ajithakumari, S. Nutritional Assessment of Tribal Women in Kainatty, Wayanad: A Cross-Sectional Study. *Indian J Community Med* **2019**, *44*, S50–S53, doi:10.4103/ijcm.IJCM_39_19.
44. Mukherjee, K.; Harihar, H.; Pulamaghatta, V.N.; Alam, A.; Rawat, B. Body Mass Index and Chronic Energy Deficiency among Adults of Tharu Population, Uttarakhand, India. *Int Jour of Biomed Res* **2015**, *6*, 475–478, doi:10.7439/ijbr.v6i7.2218.
45. Mungreiphy, N.K.; Kapoor, S. Socioeconomic Changes as Covariates of Overweight and Obesity among Tangkhul Naga Tribal Women of Manipur, North-East India. *J Biosoc Sci* **2010**, *42*, 289–305, doi:10.1017/S0021932009990587.
46. Padmavathi, C.; Ramadas, V.S. Impact of Education Programme among the Irula Tribals at Neelampathy, Ramadas V S -. *Int J Food Nutr Sci* **2012**, *1*, 42–51.
47. Raikwar, R.; Sharma, K. Health Profile and Reproductive Performance of Korku Tribal Women of Betul District, Madhya Pradesh. **2015**, *1*, 31–35.
48. James, W.P.; Ferro-Luzzi, A.; Waterlow, J.C. Definition of Chronic Energy Deficiency in Adults. Report of a Working Party of the International Dietary Energy Consultative Group. *Eur J Clin Nutr* **1988**, *42*, 969–981.
49. Sarmah, C. Ageing and Nutritional Status among Three Communities 51 of Lakhimpur District, Assam. *Indian Journal of Gerontology* **2014**, *28*, 51–69.

50. Shephard, R.J.; Shephard, R.F.; Shephard, R.J. *Body Composition in Biological Anthropology*; Cambridge University Press, 1991; ISBN 978-0-521-36267-2.
51. WHO | Global Database on Body Mass Index (BMI) Available online: https://www.who.int/nutrition/databases/bmi/en/ (accessed on 15 June 2020).
52. Sengupta, P. Capsulation of the Global Fitness Status and Body Composition of the Young Toto Women: The Smallest Tribal Community of India. *Performance Enhancement & Health* **2016**, *5*, 4–9, doi:10.1016/j.peh.2015.12.001.
53. WHO Management of Severe Malnutrition: A Manual for Physicians and Other Senior Health Workers. *Rev Panam Salud Publica* **1999**, *6*, 146–147, doi:10.1590/S1020-49891999000700020.
54. ACC/SCN *Fourth Report on the World Nutrition Situation*; ACC/SCN in collaboration with IFPRI: Geneva, 2000; p. 144.
55. Stiller, C.K.; Golembiewski, S.K.E.; Golembiewski, M.; Mondal, S.; Biesalski, H.K.; Scherbaum, V. Maternal Nutritional Status and Child Feeding Practices: A Retrospective Study in Santal Communities, Birbhum District, West Bengal, India. *Int Breastfeed J* **2020**, *15*, 50, doi:10.1186/s13006-020-00262-3.
56. Sujatha, P. Analysis of Nutrition and Reproductive Health Status of Tribal Women in Kancheepuram District. *International Journal of Research in Humanities, Arts and Science* **2017**, *2*, 95–101.
57. Toppo, S. A Comparative Study on Health Status of Oraon and Munda Rural Areas Tribal Women. *FSRJ* **2016**, *7*, 250–258, doi:10.15740/HAS/FSRJ/7.2/250-258.
58. Wani, M.; Jan, S. A Study on Nutritional and Health Status of Adult Gujjar Women of Bandipora District of Kashmir. *International Journal of Home Science* **2016**, *2*, 332–335.
59. *Minimum Dietary Diversity for Women- A Guide to Measurement*; Food and Agricultural Organization (FAO); USAID, 2016; p. 82.
60. Ghosh-Jerath, S.; Kapoor, R.; Singh, A.; Nilima, null; Downs, S.; Goldberg, G.; Fanzo, J. Agroforestry Diversity, Indigenous Food Consumption and Nutritional Outcomes in Sauria Paharia Tribal Women of Jharkhand, India. *Matern Child Nutr* **2020**, e13052, doi:10.1111/mcn.13052.
61. FAO *Minimum Dietary Diversity for Women*; FAO: Rome, Italy, 2021.
62. Ghosh-Jerath, S.; Kapoor, R.; Bandhu, A.; Singh, A.; Downs, S.; Fanzo, J. Indigenous Foods to Address Malnutrition: An Inquiry into the Diets and Nutritional Status of Women in Indigenous Community of Munda Tribes of Jharkhand, India 2022.
63. Nongrum, M.S.; Pawera, L.; Mawroh, B. Dietary Diversity and Its Determinants among Khasi and Garo Indigenous Women (15 to 49 Years) in Meghalaya, Northeast India. *Nutr Health* **2021**, 2601060211016629, doi:10.1177/02601060211016629.
