# Supplementary material for: Diet Quality, Nutritional Adequacy and Anthropometric Status among Indigenous Women of Reproductive Age Group (15–49 Years) in India: A Narrative Review
Source: Dietetics (Basel). Author manuscript; Available in PMC 2023 Aug 25. (PMC7614979; doi:10.3390/dietetics2010001)
Supplement: Table S2 [file EMS182764-supplement-Table_S2.pdf]

Table S2. Daily consumption of food group among indigenous women of India.

Tribe	N		Cereals (g/day)	Pulses (g/day)	GLVs (g/day)	Roots & Tubers (g/day)	Other vegetables (g/day)	Fruits (g/day)	Meat & Poultry (g/day)	Fish and other seafoods (g/day)	Milk & milk products (g/day)	Visible fats & oils (g/day)	Sugar & Jaggery (g/day)	Dietary assessment method	Random sample (yes/no) (g/day)
		**70% RDI[a]**	300	90	150	100	200	150		63	300	20	-		
**Jharkhand**															
Munda [1]		**Median**	288.4	22.9	41.7	56.6	52.1	8.7	37.5		ND	ND	ND	1	Yes
		**IQR**	218.3-375	12.4-41.7	25.8-63.8	31.2-98.3	26.1-93.8	3.1-23.3	22.5-72.8						
**Meghalaya**															
Khasi [2]	47	**Mean**	419.3	14.1	24.5	95.6	49.6	3.1	51.6	18.7	9.5	6.8	20.7	1	Yes
		**SD**	121.6	31	48.9	68.3	71.9	11.9	58.4	55	29.2	8.1	8.7		
**Rajasthan**															
Saharia [3]	93	**Mean**	275.2	27.9	34.9	43.5	16.7	9.8	-	-	97.1	12.8	9.0	1	No
		**SD**	59.3	24.8	45.2	31.4	29.6	23.8	-	-	44.7	5.2	4.9		
Meena [3]	92	**Mean**	276.6	20.2	38	32.6	39.4	12.2	-	-	171.2	16	10.9	1	No
		**SD**	54.6	22.3	50.2	43.9	50.3	25.8	-	-	93.5	10.2	7.2		
**Tamil Nadu**															
Irula [4]	30	**Mean**	276	61	5	91	26	91	-	-	112	30	10	2	No
Irular [5]	60	**Mean**	195	33	42	-	145	36	-	-	124	20	-	1	No

GLVs, Green leafy vegetables, RDI, Recommended dietary intake, ND, No Data. a- [6]. 1-24-hour recall method.

References

1. Ghosh-Jerath, S.; Kapoor, R.; Bandhu, A.; Singh, A.; Downs, S.; Fanzo, J. Indigenous Foods to Address Malnutrition: An Inquiry into the Diets and Nutritional Status of Women in the Indigenous Community of Munda Tribes of Jharkhand, India. *Current Developments in Nutrition* **2022**, *6*, nzac102, doi:10.1093/cdn/nzac102.
2. Chyne, D.A.L.; Meshram, I.I.; Rajendran, A.; Kodali, V.; Getti, N.; Roy, P.; Kuhnlein, H.V.; Longvah, T. Nutritional Status, Food Insecurity, and Biodiversity among the Khasi in Meghalaya, North-East India. *Matern Child Nutr* **2017**, *13 Suppl 3*, doi:10.1111/mcn.12557.
3. Chishty, S.; Singh, N. Nutritional Status of Tribal Women (Saharia and Meena), Baran District of Rajasthan, India. *Nutrition & Food Science* **2018**, *48*, doi:10.1108/NFS-01-2018-0018.
4. Padmavathi, C.; Ramadas, V.S. Impact of Education Programme among the Irula Tribals at Neelampathy, Ramadas V S -. *Int J Food Nutr Sci* **2012**, *1*, 42–51.
5. Sujatha, P. Analysis of Nutrition and Reproductive Health Status of Tribal Women in Kancheepuram District. *International Journal of Research in Humanities, Arts and Science* **2017**, *2*, 95–101.
6. ICMR-NIN *Nutrient Requirements for Indians: A Report of the Expert Group, 2020*; ICMR-National Institute of Nutrition, Indian Council of Medical Research, Department of Health Research, Ministry of Health and Family Welfare, Government of India: Hyderabad, India, 2020.
