# Supplementary material for: Diet Quality, Nutritional Adequacy and Anthropometric Status among Indigenous Women of Reproductive Age Group (15–49 Years) in India: A Narrative Review
Source: Dietetics (Basel). Author manuscript; Available in PMC 2023 Aug 25. (PMC7614979; doi:10.3390/dietetics2010001)
Supplement: Table S3 [file EMS182764-supplement-Table_S3.pdf]

Table S3. Dietary diversity scores in indigenous women of India.

Indigenous community (state)	Mean MDD-W score
Sauria Paharia (Jharkhand)	3 (median)
Munda (Jharkhand)	2.6 (monsoon); 3 (winters)
Khasi (Meghalaya)	3.7
Garo (Meghalaya)	4.8
